# Estimating the COVID-19 Spread Through Real-time Population Mobility Patterns: Surveillance in Low- and Middle-Income Countries

**DOI:** 10.2196/22999

**Published:** 2021-06-14

**Authors:** Stefanos Tyrovolas, Iago Giné-Vázquez, Daniel Fernández, Marianthi Morena, Ai Koyanagi, Mark Janko, Josep Maria Haro, Yang Lin, Paul Lee, William Pan, Demosthenes Panagiotakos, Alex Molassiotis

**Affiliations:** 1 School of Nursing Hong Kong Polytechnic University Kowloon Hong Kong; 2 Parc Sanitari Sant Joan de Déu Universitat de Barcelona, Fundació Sant Joan de Déu Barcelona Spain; 3 Instituto de Salud Carlos III Centro de Investigación Biomédica en Red de Salud Mental Madrid Spain; 4 Nutrition and Dietetics Department School of Health Science and Education, Harokopio University Athens Greece; 5 Department of Statistics and Operations Research Polytechnic University of Catalonia (UPC)-BarcelonaTech Barcelona Spain; 6 Institute of Mathematics of Polytechnic University of Catalonia (UPC)-BarcelonaTech (IMTech) Barcelona Spain; 7 Catalan Institution for Research and Advanced Studies Barcelona Spain; 8 Duke University Durham, NC United States; 9 Department of Health Sciences University of Leicester Leicester United Kingdom

**Keywords:** COVID-19, transmission, digital public health, social distancing, policy, mobile data, estimate, real-time, pattern, surveillance, low and middle-income countries, emerging countries, database, surveillance

## Abstract

**Background:**

On January 21, 2020, the World Health Organization reported the first case of severe acute respiratory syndrome coronavirus 2, which rapidly evolved to the COVID-19 pandemic. Since then, the virus has also rapidly spread among Latin American, Caribbean, and African countries.

**Objective:**

The first aim of this study is to identify new emerging COVID-19 clusters over time and space (from January 21 to mid-May 2020) in Latin American, Caribbean, and African regions, using a prospective space–time scan measurement approach. The second aim is to assess the impact of real-time population mobility patterns between January 21 and May 18, 2020, under the implemented government interventions, measurements, and policy restrictions on COVID-19 spread among those regions and worldwide.

**Methods:**

We created a global COVID-19 database, of 218 countries and territories, merging the World Health Organization daily case reports with other measures such as population density and country income levels for January 21 to May 18, 2020. A score of government policy interventions was created for *low*, *intermediate*, *high*, and *very high* interventions. The population’s mobility patterns at the country level were obtained from Google community mobility reports. The prospective space–time scan statistic method was applied in five time periods between January and May 2020, and a regression mixed model analysis was used.

**Results:**

We found that COVID-19 emerging clusters within these five periods of time increased from 7 emerging clusters to 28 by mid-May 2020. We also detected various increasing and decreasing relative risk estimates of COVID-19 spread among Latin American, Caribbean, and African countries within the period of analysis. Globally, population mobility to parks and similar leisure areas during at least a minimum of implemented intermediate-level control policies (when compared to low-level control policies) was related to accelerated COVID-19 spread. Results were almost consistent when regional stratified analysis was applied. In addition, worldwide population mobility due to working during high implemented control policies and very high implemented control policies, when compared to low-level control policies, was related to positive COVID-19 spread.

**Conclusions:**

The prospective space–time scan is an approach that low-income and middle-income countries could use to detect emerging clusters in a timely manner and implement specific control policies and interventions to slow down COVID-19 transmission. In addition, real-time population mobility obtained from crowdsourced digital data could be useful for current and future targeted public health and mitigation policies at a global and regional level.

## Introduction

On January 30, 2020, the World Health Organization (WHO) declared COVID-19 as a Public Health Emergency of International Concern and later characterized it as a pandemic [1]. On January 21, 2020, the WHO published the first situation report, announcing the first cases of pneumonia of unknown etiology detected in Wuhan City on December 31, 2019 [2]. By May 18, 2020, more than 200 countries reported confirmed cases of COVID-19 [2,3]. Among them, there were several Latin American, Caribbean, and African countries with limited resources to monitor, manage, and treat COVID-19. The first signs of virus spread were delayed among these regions compared with Europe, Asia, and North America [2,4].

Several government interventions have already been implemented to prevent and contain the alarming propagation of COVID-19 [5,6]. Each country has applied its own disease control measures, which vary by specific policy and implementation timing. Some countries initially implement a *lower* level of measures and policies, while others are adopting *stricter* ones. Government health and social distancing policies are evolving rapidly based on the COVID-19 transmission in each region. Policies range from traveler’s temperature checks and medical screening at each country’s entry point and public school closures to quarantining an entire country. Various Latin American and African countries adopted COVID-19 restriction policies rapidly [7]. Barriers such as the effectiveness of social distancing measures among low-income and middle-income countries (LMICs) have been pointed out [8]. For these reasons, global and local health policy makers and international organizations have said that the lack of health and government resources among these regions would pose barriers and challenges to halt virus spread.

Space–time surveillance is a methodology [9] that could be of use among Latin American and African regions to identify and list locations in an emergency, apply the strictest public health measures, and allocate resources. The space–time scan statistic technique is able to detect *dynamic* or emerging clusters of COVID-19, which can be used for targeted monitoring during the outbreak [10]. Since COVID-19 data are updated daily, this method could contribute to timely monitoring of the pandemic among various areas such as Latin American and African regions. In addition to statistical approaches, digital technology [11,12] could be used to understand population mobility and to assess the effectiveness of government policies or the re-evaluation of specific strategies. During the COVID-19 outbreak, smartphone software can provide information (in an anonymous way and at the country level) [13] on various characteristics of population mobility (eg, workplaces or parks). This information could be of use among countries and specifically in resource-limited settings to understand rapidly whether the government restrictions need enhancements or corrections.

In the early stage of the COVID-19 spread in the United States, prospective scan statistic methodology detected the *active* cluster in New York State, marking the area that needed specific attention [10]. To the best of our knowledge during COVID-19, except in the United States, the prospective scan method has not been applied elsewhere, though it could be a useful monitoring and intervention–decision tool. In addition, the collective effect sizes of population mobility patterns under the social distancing government policies are empirically unknown, particularly in low-income countries (LICs) and middle-income countries (MICs) with differential population vulnerability (ie, poverty, lack of resources, and health infrastructure). Given the information regarding effective treatment schemes and population vaccination going forward slowly and taking into account the delays in deliveries among countries, detection of emerging clusters among these regions will make a substantial contribution [14] to the field facilitating the translation of knowledge and implementation of evidence into COVID-19 practice and policy at the country level. In addition, it will guide authorities globally and among low- and middle-income countries (LMICs) to enhance and update if necessary the applied COVID-19 containment policies based on real-time population mobility. The first aim of this study, therefore, is to identify new emerging space–time COVID-19 clusters implementing space–time surveillance among Latin American, Caribbean, and African countries, applying a prospective space–time scan statistic methodology. This technique is a well-known method for detecting clusters of health-related events in the space–time dimension [10]. Our available data extends until May 18, 2020. Thus, we report results applying the prospective space–time scan statistic in five time periods to monitor the emerging clusters when adding updated case data: (1) January 21 to March 15, (2) January 21 to March 31, (3) January 21 to April 15, (4) January 21to April 30, and (5) January 21 to May 15. The second aim is to assess the impact of real-time population mobility patterns between January 21 and May 18 under the implemented government interventions, measurements, and policy restrictions on COVID-19 spread among Latin American, Caribbean, and African countries as well as globally. This study focuses its analysis on Latin American, Caribbean, and African countries (as a sample of LMICs) among other Asian-Pacific regions due to the excessive COVID-19 transmission in these areas. This study could serve as a learning tool presenting new information on virus surveillance and its timely detection among countries and regions with limited resources at their disposal, while population mobility patterns will facilitate public health authorities to design targeted social distancing strategies instead of *horizontal* lockdowns or social distancing measures. Results of this study in combination with lessons from other countries’ experiences [15] could be helpful for policy makers at regional and international level.

## Methods

### Study Design

We conducted a retrospective observational longitudinal study. We obtained data on COVID-19 propagation and related risk factors from 218 countries and territories (as reported by the WHO). We compiled a data set of COVID-19 daily cases and deaths spanning the period January 21 to May 18, 2020, based on the most recent publicly available population-level information (by country), as reported by the WHO [2]. This study was approved by Parc Sanitari Sant Joan de Déu, Ethics Committee (PIC-67-20, Barcelona, Spain) and conformed to the ethical guidelines of the 1975 Declaration of Helsinki.

### COVID-19 International Data

The WHO daily situation reports were used from January 21 to May 18, 2020, to assess disease transmission internationally [2]. Data on daily confirmed cases, total confirmed cases, daily confirmed deaths, total confirmed deaths, transmission classification, and time since the last reported case were compiled for 218 countries and territories. Case classifications were based on the WHO case definitions for COVID-19. Transmission was classified into three distinct groups to capture changes in the classification that the WHO applied during these 4 months: community transmission, transmission under investigation, and sporadic clusters transmission (includes sporadic transmission, clusters, and local transmission) [2]. Cases identified in cruise ships were excluded from the analysis, while cases among China’s provinces were grouped together. Cases in Hong Kong, Macao, and Taiwan special administrative regions of China were classified separately since they applied different government interventions and policy measures than mainland China. Based on the WHO database, Puerto Rico was classified separately from the United States as was the case for other territories.

### Countries’ Government Interventions, Health Policy, and Restriction Measures

Each country’s health and government policy measures were obtained as announced from each country’s official source after January 21, 2020. If this was not feasible, the information was obtained from local media sources and was cross-checked with at least two sources (where possible). Additionally, two researchers cross-validated the obtained information to ensure the highest accuracy. This information was then validated using the WHO global tracking database on governments’ COVID-19 response as the gold standard database [16]. Based on this information, a four-level health and government policy interventions and measures score was created, ranging from 0 to 3, which represented *low*, *intermediate*, *high*, and *very high* intervention levels [17]. These intervention and control policy categories were formed following already announced alert classification systems [18] and other international COVID-19 government response data and methodologies [19,20].

### Other Baseline Assessments by Country

#### Index for Risk Management and World Bank Income Classification

Information regarding threat detection and risk assessment were obtained from the Index for Risk Management (INFORM) Epidemic Risk Index [21], developed by the EU Joint Research Centre in collaboration with the WHO, and was used as a measure of each country’s epidemic preparedness. The INFORM index ranged from 0 to 10, and higher scores corresponded to a lower epidemic preparedness risk of a country. More detail about the development of this index can be found elsewhere [21].

The World Bank income classification system was also used to classify each of the 218 countries’ income (high-income countries, upper-income countries, lower middle–income countries, and low-income countries) [22]. COVID-19 testing policy in each country was assessed as the number of days that each country started implementing COVID-19 tests in the population and as the number of days that each country implemented tracing strategies for COVID-19 cases. Information on these items was obtained from publicly available sources [20].

#### Cell Phones and Community Mobility Reports

The population’s mobility patterns at the country level were obtained from Google community mobility reports. These reports are publicly available [23] and present movement trends over time by geography and across different place categorization such as retail and recreation places, groceries and pharmacies, parks and other similar places, transit stations, workplaces, and residential areas. Specifically, as described by Google reports, retail and recreation grouping correspond to mobility trends for places such as restaurants, cafés, shopping centers, theme parks, museums, libraries, and movie theaters. Groceries and pharmacies reports mobility trends for places such as grocery markets, food warehouses, farmers markets, specialty food shops, drug stores, and pharmacies. The parks category encompasses mobility trends for places such as national parks, public beaches, marinas, dog parks, plazas, and public gardens. In addition, transit station cluster marks mobility trends for places such as public transport hubs (eg, subway, bus, and train stations). The workplace classification corresponds to the mobility trend for places of work. Finally, the residential cluster encompasses mobility trends for staying at home. These reports show how visits and length of stay at different places change compared to a specific reference period (a reference period defined by Google as, for example, January 3 to February 6, 2020). Data in these reports are generated using aggregated anonymized sets of data from users that turned on the location history setting.

Of the 218 countries and territories, 179 had complete data and were selected for the adjusted analysis. In this analysis, only COVID-19 daily new cases were analyzed. Analysis was applied globally only between real-time population mobility patterns and COVID-19 spread, while the rest of the analysis was restricted for the regions of Latin America, the Caribbean, and Africa. By May 15, 2020, we calculated the standardized incidence ratios (SIRs) [24] for 4 countries (Brazil, Peru, Uganda, and Nigeria) to compare and validate the accuracy of our prospective space–time models. COVID-19 SIR estimations were calculated as the ratio of observed counts to the expected counts.

### Statistical Analysis

Based on the literature review, government interventions are not having an immediate effect on COVID-19 spread; for this reason, we considered their time scheduling based on a starting time point t0, with the addition of a seven-day lag [25]. These time lag effects only concern the modeling process via a mixed model approach, as it is when our analysis tested the government control policies.

#### Prospective Space–Time Scan Statistic

The early detection of emerging COVID-19 space–time clusters was determined using a prospective version of the space–time scan statistic approach [9]. The method helps to identify COVID-19 clusters in the space–time dimension, which have a significant relative risk (RR) at the end of the study period [10]. The general assumption is that the number of COVID-19 cases follows a Poisson distribution with a constant risk, which is proportional to the at-risk population of each corresponding country or territory over the geographic region under study.

This approach works using cylinders that move and scan the region of interest looking for potential space–time clusters of COVID-19 cases. The center of the cylinder is defined as the centroid of each country in the region of interest. The general working function of this technique can be summarized as follows: an unknown large number of cylinders of different spatial and temporal sizes are generated around each centroid until the maximum spatial and temporal thresholds are reached; the observed and expected case counts are computed within each cylinder, which is derived from the total number of centroids captured in each cylinder.

In this manner, the RR is defined as having more observed than expected COVID-19 cases within each cylinder. We determined the elevated RR of COVID-19 calculating maximum log-likelihood ratio tests. Furthermore, 100 runs of Monte Carlo testing were used to depict the empirical distribution of the log-likelihood ratio, assuming constant risk. This distribution allows us to assess the statistical significance of space–time clusters (*P* value <.05), and the cylinder with the largest log-likelihood ratio is the most likely cluster.

In our study, we reported the significant emerging clusters of COVID-19 at the country level for the Latin American, Caribbean, and African regions, and computed the estimated RR, which identifies the risk for the population to COVID-19 within a cluster in comparison with the risk outside of the cluster. Moreover, five time periods were monitored: (1) January 21 to March 15; (2) January 21 to March 31; (3) January 21 to April 15; (4) January 21 to April 30; and (5) January 21 to May 15, 2020.

This analysis was carried out using the R package *scanstatistics* in R version 3.6.3 (R Foundation for Statistical Computing) and follows previous prospective scan statistic work [9,10,26].

#### Mixed Models Analysis

We also fitted a negative binomial (NB) mixed model, with daily new COVID-19 cases as the outcome. The model accounts for a linear trend with respect to time since the appearance of the first COVID-19 case, taking into account the varying secular trends across regions and the treatment–effect heterogeneity across time, and was adjusted for the countries’ preparedness in epidemics (INFORM index), COVID-19 testing policy, COVID-19 type of transmission for each country through time, populations’ real-time mobility patterns, their interaction with each level of government control policy, and the country’s income level. In this mixed model, all the predictors are assumed to be fixed effects; however, the intercept includes a country-level random effect term. As offset, the natural logarithm of the total population was added to the generalized linear predictor function of the NB component to account for the variable number of daily new COVID-19 cases per country population. Models were tested globally and regionally (for Latin American, Caribbean, and African countries). The maximum likelihood estimation procedure was used to fit all multilevel analysis models. In this mixed model, only three out of five real-time mobility patterns for workplaces, parks, and similar places as well as mobility for food and drug supplies were applied to avoid collinearity, as the correlation among the mobility variables was higher than 0.70. Mixed model analysis was carried out using the R package g*lmmTMB* in R Version 3.6.3 [27].

#### Validation Analysis

First, the predicted validity of the prospective scan statistic model was tested evaluating the relation between the RRs of the prospective scan statistic and the SIR estimations using Pearson rho coefficients. Second, we used the Man-Whitney *U* test to explore the increase in mean COVID-19 cases before and after April 15, 2020, and then, we checked if the prospective scan had predicted a potential COVID-19 cluster. We followed classical reported criteria to classify a correlation as weak (≤0.3), moderate (0.4-0.6), and strong (≥0.7; coefficients are presented as absolute values) [28]. All *P* values are based on two-sided tests. A *P* value ≤.05 was considered as significant.

## Results

### Prospective Scan Statistics and Emerging Country-Level Results Between January 21 and May 15 for Latin America, the Caribbean, and Africa

Tables 1 and 2 provide the characteristics of the significant COVID-19 emerging space–time clusters at the country level among LMICs, from January 21 to May 15, 2020. Analyzing COVID-19 spread between January 21 to March 15, 7 major clusters among Latin American, Caribbean, and African countries were revealed. For the Latin American and Caribbean countries, cluster 1 included 15 countries with a RR>1 (ie, having more observed than expected COVID-19 cases). Saint Kitts and Nevis (cluster 3) showed an extremely elevated RR of 19.31 (*P*<.001). Exploring the African region during the same period, cluster 4 integrated most of the countries with a RR of 9.46 (*P*<.001), and cluster 5 encompassed Madagascar, Mauritius, Mayotte, Reunion, and Seychelles with a RR of 21.35 (*P*<.001). Rwanda and Uganda were grouped in cluster 6 and Sudan in cluster 7. Both these clusters marked the most elevated RR (cluster 6: RR 37.75, *P*<.001; cluster 7, RR 45.75, *P*<.001; Figures S1 and S2 in Multimedia Appendix 1).

Using data for the period of January 21 to March 31, 2020, allowed us to assess the evolution of COVID-19 spread among LMICs. It was shown that the initial 7 emerging COVID-19 country clusters among Latin America, the Caribbean, and Africa, when the period of analysis extended for 15 days, were spread to 20 clusters. Cluster 5 that included only Antigua and Barbuda Island had the most elevated relative risk (RR 60.48; *P*<.001) followed by cluster 6 (Puerto Rico, Saint Maarten, and Virgin Islands) with a RR of 23.27 (*P*<.001) and cluster 8 (Dominica) with a RR of 21.85 (*P*<.001; Figures S3 and S4 in Multimedia Appendix 1).

Analyzing data from January 21 to April 15-30 and to May 15, 2020, it was shown that the spread was further extended with the evolution of time. Specifically, when the period of analysis extended to April 15, the virus spread was reported to 27 clusters among Latin America, the Caribbean, and Africa, and by April 30 and May 15, the emerging clusters were 29 and 28, respectively. For the period between January 21 to April 15 for Latin America and the Caribbean, the cluster with the highest RR was Chile (RR 18.02; *P*<.001), followed by Antigua and Barbuda (RR 14.22; *P*<.001), and Mexico (RR 14.07; *P*<.001). For Africa, the emerging clusters were Djibouti (RR 165.84; *P*<.001), followed by Mauritius (RR 136.05; *P*<.001) and Egypt (RR 52.20; *P*<.001). Focusing on the period between January 21 to April 30 and to May 15, we showed extended COVID-19 spread following similar patterns as previously mentioned (Figures S5-S10 in Multimedia Appendix 1).

**Table 1:** Emerging COVID-19 space–time clusters and their RR for having more observed than expected COVID-19 cases, from January 21 to March 15 and March 31, 2020, at the country level within the Latin American, Caribbean, and African regions.

Region, date range, and cluster number				Cluster	Duration		RR^a,b^
**Latin America and Caribbean**							
	**January 21 to March 15**						
		1	Argentina, Bolivia, Brazil, Barbados, Colombia, Grenada, Guyana, Saint Lucia, Peru, Paraguay, Suriname, Trinidad, Uruguay, Saint Vincent, Venezuela		March 11-15, 2020	3.02	
		2	Bahamas, Belize, Costa Rica, Cuba, Cayman Islands, Ecuador, Guatemala, Honduras, Haiti, Jamaica, Mexico, Nicaragua, Panama, El Salvador, Turks and Caicos Islands		March 9-15, 2020	4.04	
		3	Saint Kitts and Nevis		March 12-15, 2020	19.31	
	**January 21 to March 31**						
		1	Bolivia, Colombia, Ecuador, Peru		March 17-31, 2020	11.61	
		2	Bahamas, Belize, Costa Rica, Cuba, Cayman Islands, Dominican Republic, Guatemala, Honduras, Haiti, Jamaica, Mexico, Nicaragua, Panama, El Salvador, Turks and Caicos Islands		March 22-31, 2020	3.87	
		3	Barbados, Grenada, Trinidad, Saint Vincent		March 22-31, 2020	2.53	
		4	Argentina, Chile, Paraguay, Uruguay		March 24-31, 2020	2.79	
		5	Antigua and Barbuda		March 21-31, 2020	60.48	
		6	Puerto Rico, Saint Maarten, Virgin Islands		March 20-31, 2020	23.27	
		7	Curacao		March 27-31, 2020	2.99	
		8	Dominica		March 22-31, 2020	21.85	
		9	Saint Lucia		March 26-31, 2020	1.25	
**Africa**							
	**January 21** **to March 15**						
		4	Benin, Burkina Faso, Ivory Coast, Algeria, Ghana, Guinea, Gambia, Mali, Mauritania, Senegal, Sierra Leone, Togo		March 6-15, 2020	9.46	
		5	Madagascar, Mauritius, Mayotte, Reunion, Seychelles		March 13-15, 2020	21.35	
		6	Rwanda, Uganda		March 9-15, 2020	37.75	
		7	Sudan		March 13-15, 2020	45.75	
	**January 21 to March 31**						
		10	Madagascar, Mozambique, Mauritius, Mayotte, Reunion, Seychelles		March 17-31, 2020	8.75	
		11	Rwanda, Uganda		March 18-31, 2020	34.34	
		12	Benin, Burkina Faso, Ivory Coast, Algeria, Ghana, Guinea, Gambia, Mali, Mauritania, Senegal, Sierra Leone, Togo		March 19-31, 2020	3.83	
		13	Botswana		March 15-31, 2020	6.92	
		14	Equatorial Guinea		March 21-31, 2020	5.91	
		15	Djibouti		March 22-31, 2020	31.00	
		16	Egypt, Sudan		March 20-31, 2020	4.47	
		17	Guinea-Bissau		March 30-31, 2021	3.27	
		18	Zambia		March 26-31, 2020	13.84	
		19	Tanzania		March 18-31, 2020	2.85	
		20	Morocco		March 28-31, 2021	2.86	

^a^RR: relative risk estimate.

^b^All RRs have a *P* value <.001

**Table 2:** Emerging COVID-19 space–time clusters and their RR for having more observed than expected COVID-19 cases from January 21 to April 15, April 30 and May 15, 2020, at the country level within the Latin American, Caribbean, and African regions.

Region, date range, and cluster number				Cluster		Duration		RR^a,b^
**Latin America and Caribbean**								
	**January 21 to April 15**							
		1	Colombia, Costa Rica, Ecuador, Panama, Peru		March 29-April 15, 2020		5.27	
		2	Barbados		March 31-April 15, 2020		2.81	
		3	Mexico		March 21-April 15, 2020		14.07	
		4	Dominican Republic		March 22-April 15, 2020		5.94	
		5	Puerto Rico, Saint Maarten, Virgin Islands		March 22-April 15, 2020		9.88	
		6	Antigua and Barbuda		March 21-April 15, 2020		14.22	
		7	Saint Vincent		March 17-April 15, 2020		2.08	
		8	Chile		March 29-April 15, 2020		18.02	
		9	Curacao		April 6-15, 2020		1.94	
		10	Belize, Guatemala, El Salvador		March 22-April 15, 2020		4.54	
		11	Bahamas, Cuba		March 18-April 15, 2020		1.74	
		12	Saint Lucia		April 10-15, 2020		1.33	
		13	Argentina, Uruguay		April 12-15, 2020		1.61	
		14	Saint Kitts and Nevis		April 14-15, 2020		4.66	
		15	Dominica		March 22-April 15, 2020		3.98	
	**January 21 to April 30**							
		1	Colombia, Ecuador, Panama, Venezuela		April 7-30, 2020		5.85	
		2	Barbados		April 15-30, 2020		3.44	
		3	Mexico		March 21-April 30, 2020		7.64	
		4	Dominican Republic		March 29-April 30, 2020		3.84	
		5	Antigua and Barbuda, Dominica, Saint Kitts and Nevis, Saint Lucia		April 22-30, 2020		1.67	
		6	Peru		March 29-April 30, 2020		2.95	
		7	Saint Maarten		March 29-April 30, 2020		11.47	
		8	Chile		March 29-April 30, 2020		6.47	
		9	Virgin Islands		March 20-April 30, 2020		2.94	
		10	El Salvador		March 17-April 30, 2020		4.49	
		11	Costa Rica		April 28-30, 2020		1.26	
	**January 21 to May 15**							
		1	Bolivia, Brazil, Barbados, Colombia, Grenada, Guyana, Peru, Paraguay, Suriname, Trinidad, Uruguay, Venezuela		April 25-May 15, 2020		3.40	
		2	Panama		April 14-May 15, 2020		8.28	
		3	Ecuador		April 7-May 15, 2020		5.32	
		4	Mexico		March 24-May 15, 2020		4.54	
		5	Dominican Republic		April 8-May 15, 2020		2.80	
		6	Saint Lucia		May 6-15, 2020		1.64	
		7	Costa Rica		May 8-15, 2020		1.34	
		8	Saint Maarten		March 29-May 15, 2020		4.41	
		9	Jamaica		May 4-15, 2020		1.35	
		10	Chile		March 29-May 15, 2020		3.17	
		11	Antigua and Barbuda		March 21-May 15, 2020		1.85	
**Africa**								
	**January 21-April 15**							
		16	Botswana, Mozambique, Malawi, Swaziland, Zambia, Zimbabwe		March 22-April 15, 2020		5.91	
		17	Benin, Burkina Faso, Ivory Coast, Algeria, Ghana, Guinea, Gambia, Guinea-Bissau, Liberia, Mali, Mauritania, Niger, Senegal, Sierra Leone, Togo		March 31-April 15, 2020		5.54	
		18	Rwanda, Uganda		March 19-April 15, 2020		15.17	
		19	Djibouti		April 4-15, 2020		165.84	
		20	Mauritius		March 20-April 15, 2020		136.05	
		21	Gabon, Equatorial Guinea		March 23-April 15, 2020		5.17	
		22	Reunion		April 14-15, 2020		37.35	
		23	Egypt		April 13-15, 2020		52.20	
		24	Sao Tome and Principe		April 12-15, 2020		5.56	
		25	Libya, Tunisia		April 14-15, 2020		3.47	
		26	Somalia		March 14-15, 2020		16.18	
		27	Sudan		March 22-April 15, 2020		1.84	
	**January 21 to April 30**							
		12	Benin, Burkina Faso, Ivory Coast, Algeria, Ghana, Guinea, Gambia, Guinea-Bissau, Liberia, Mali, Mauritania, Niger, Senegal, Sierra Leone, Togo		March 31-April 30, 2020		4.26	
		13	Comoros, Mozambique, Malawi, Swaziland, Zambia, Zimbabwe		March 22-April 30, 2020		4.85	
		14	Djibouti		April 5-30, 2020		108.36	
		15	Mauritius		March 20-April 30, 2020		117.76	
		16	Cameroon, Gabon, Equatorial Guinea, Sao Tome and Principe		April 5-30, 2020		4.71	
		17	Rwanda, Uganda		March 19-April 30, 2020		6.57	
		18	Egypt		April 13-30, 2020		33.32	
		19	Nigeria		April 28-30, 2020		3.83	
		20	Tunisia		April 28-30, 2020		4.03	
		21	Reunion		April 11-30, 2020		4.05	
		22	Botswana		March 20-April 30, 2020		1.88	
		23	Chad		April 24-30, 2020		2.69	
		24	Democratic Republic of the Congo		April 26-30, 2020		6.08	
		25	South Sudan		April 16-30, 2020		2.57	
		26	Sudan		April 23-30, 2020		2.61	
		27	Cape Verde		April 29-30, 2020		2.16	
		28	Republic of Kong		April 29-30, 2020		3.42	
		29	Somalia		March 14-April 30, 2020		6.10	
	**January 21-May 15**							
		12	Benin, Burkina Faso, Ivory Coast, Algeria, Ghana, Guinea, Gambia, Guinea-Bissau, Liberia, Mali, Mauritania, Niger, Senegal, Sierra Leone, Togo		April 3-May 15, 2020		4.84	
		13	Comoros, Mozambique, Malawi, Swaziland, Zambia, Zimbabwe		April 11-May 15, 2020		7.35	
		14	Mauritius		March 20-May 15, 2020		151.42	
		15	Djibouti		April 4-May 15, 2020		60.47	
		16	Cameroon, Gabon, Equatorial Guinea, Sao Tome and Principe		April 12-May 15, 2020		5.65	
		17	Egypt		April 13-May 15, 2020		25.85	
		18	Nigeria		April 28-May 15, 2020		2.33	
		19	Chad		April 28-May 15, 2020		4.38	
		20	Rwanda, Uganda		March 19-May 15, 2020		3.79	
		21	Democratic Republic of the Congo		May 8-15, 2020		11.98	
		22	South Sudan, Tanzania		May 3-15, 2020		4.35	
		23	Reunion		April 14-May 15, 2020		3.68	
		24	Republic of Congo		May 4-15, 2020		3.45	
		25	Seychelles		May 6-15, 2020		3.31	
		26	Cape Verde		May 5-15, 2020		1.49	
		27	Libya		April 7-May 15, 2020		2.12	
		28	Botswana		March 20-May 15, 2020		1.26	

^a^RR: relative risk estimate.

^b^All RRs have a *P* value <.001.

#### Predictive Validity of the Prospective Scan Model

To evaluate the predictive validity of the prospective scan statistic approach, we used Brazil, Peru, Uganda, and Nigeria as country examples. Figure S11 in Multimedia Appendix 1 illustrates the RRs of the prospective scan approach and the SIR estimations. The correlation of both models for all 4 countries was strong and significant (Uganda: ρ=0.78, *P*=.04; Nigeria: ρ=0.98, *P*=.002; Brazil: ρ=0.95, *P*=.01; and Peru: ρ=0.86, *P*=.03). In addition, among all 4 countries that prospective scan modelling predicted a potential outbreak after April 15, 2020, we found a significant increase in the comparison of the COVID-19 mean new cases before and after April 15 (Uganda: W=697, *P*=.008; Nigeria: W=1280, *P*<.001; Brazil: W=1596, *P*<.001; and Peru: W=1246, *P*<.001).

### COVID-19 Spread in Relation to Real-time Population Mobility Patterns Between January 21 and May 18 Globally and Regionally for Latin America, the Caribbean, and Africa

#### Population Mobility Patterns and COVID-19 Spread at the Global Level

COVID-19 daily new cases and real-time population mobility changes by region are presented in Figure S12 in Multimedia Appendix 1. Among the 3 population mobility patterns, a reduced change in comparison with the reference period of time was observed. Population mobility patterns and COVID-19 spread worldwide and by the Latin American, Caribbean, and African region are reported in Table 3. Worldwide, between January and May 2020, population mobility to all kinds of food places, drug stores, and pharmacies was not associated with COVID-19 spread, while the use of park places (ie, national and city parks, public beaches, and dog parks) and mobility to workplaces were negatively related with COVID-19 spread (mobility to parks and similar places: *b*=–0.03, 95% CI –0.04 to –0.02); workplaces mobility: *b*=–0.03, 95% CI –0.05 to –0.02). However, when the interaction effect between government control policies (intermediate, high, and very high) and population’s mobility patterns was applied and compared with that of low-level government control policies, different trends were extracted regarding COVID-19 spread. It was observed that COVID-19 spread changes significantly throughout mobility confounders depending on the degree of the implemented control policies. Specifically, with the implementation of intermediate, high, and very high control policies, mobility to parks and other similar places (like beaches, dog parks, and others) were related with increased COVID-19 spread (*b*=0.02, 95% CI 0.01-0.03; high-level interventions: *b*=0.02, 95% CI 0.01-0.03; very high–level interventions: *b*=0.02, 95% CI 0.01-0.03) when compared with the population mobility in parks during the implementation of low-level government control policies. Similar increased COVID-19 spread estimates were shown for population mobility to workplaces during high-level and very high–level movement restrictions (high-level interventions: *b*=0.02, 95% CI 0.01-0.04; very high–level interventions: *b*=0.03, 95% CI 0.01-0.04) when compared with population mobility to workplaces during the implementation of low-level government policies.

**Table 3:** Results from mixed model analysis that evaluated the COVID-19 spread with government interventions, their interaction with population mobility patterns, and other factors during the first 4 months of the outbreak.^a^

Items	Global, *b* (95% CI)	Latin America, the Caribbean, and Africa, *b* (95% CI)		Latin America and the Caribbean, *b* (95% CI)		Africa, *b* (95% CI)	
Number of days since first case	–0.003 (–0.01 to 0.01)	0.03 (0.00^b^ to 0.06)	0.09 (0.05 to 0.13)		0.02 (–0.05 to 0.09)		
Low-level interventions (reference category)	N/A^c^	N/A	N/A		N/A		
Intermediate-level interventions	–0.90 (–1.23 to –0.56)	0.48 (–0.62 to 1.58)	2.42 (–1.50 to 6.35)		0.41 (–1.20 to 2.03)		
High-level interventions	0.88 (0.59 to 1.16)	0.92 (0.35 to 1.49)	2.42 (1.52 to 3.32)		0.90 (–0.19 to 1.98)		
Very high–level interventions	0.74 (0.40 to 1.08)	0.27 (–0.39 to 0.94)	1.94 (0.73 to 3.16)		0.25 (–0.88 to 1.39)		
Population mobility for food and drug supplies	0.004 (–0.02 to 0.02)	–0.01 (–0.05 to 0.03)	–0.004 (–0.05 to 0.05)		–0.03 (–0.10 to 0.05)		
Population mobility to parks/leisure activities	–0.03 (–0.04 to –0.02)	–0.07 (–0.10 to –0.05)	–0.10 (–0.13 to –0.07)		–0.04 (–0.10 to 0.02)		
Population mobility to workplace	–0.03 (–0.05 to –0.02)	–0.02 (–0.04 to 0.00^b^)	–0.003 (–0.02 to 0.01)		–0.05 (–0.09 to –0.01)		
Number of days since first case × low-level interventions (reference category)	N/A	N/A	N/A		N/A		
Number of days since first case × intermediate-level interventions	0.04 (0.03 to 0.05)	–0.02 (–0.07 to 0.03)	–0.28 (–0.92 to 0.36)		0.0004 (–0.09 to 0.09)		
Number of days since first case × high-level interventions	–0.001 (–0.01 to 0.01)	0.002 (–0.03 to 0.03)	–0.06 (–0.10 to –0.02)		0.01 (–0.06 to 0.08)		
Number of days since first case × very high–level interventions	0.005 (–0.01 to 0.01)	–0.001 (–0.03 to 0.03)	–0.07 (–0.11 to –0.02)		0.006 (–0.06 to 0.07)		
Low-level intervention × population mobility for food and drug supplies (reference category)	N/A	N/A	N/A		N/A		
Intermediate-level intervention × population mobility for food and drug supplies	0.02 (–0.00 to 0.04)	0.02 (–0.05 to 0.09)	0.01 (–0.08 to 0.11)		0.05 (–0.09 to 0.18)		
High-level intervention × population mobility for food and drug supplies	0.004 (–0.02 to 0.02)	0.03 (–0.00^b^ to 0.07)	0.03 (–0.03 to 0.08)		0.03 (–0.05 to 0.11)		
Very high–level intervention × population mobility for food and drug supplies	0.001 (–0.02 to 0.02)	0.02 (–0.02 to 0.05)	–0.003 (–0.05 to 0.05)		0.05 (–0.03 to 0.12)		
Low-level intervention × population mobility to visit parks and do leisure activities (reference category)	N/A	N/A	N/A		N/A		
Intermediate-level intervention × population mobility to visit parks and do leisure activities	0.02 (0.01 to 0.03)	0.03 (–0.01 to 0.06)	–0.02 (–0.28 to 0.23)		–0.02 (–0.10 to 0.06)		
High-level intervention × population mobility to visit parks and do leisure activities	0.02 (0.01 to 0.03)	0.05 (0.02 to 0.07)	0.07 (0.04 to 0.10)		0.04 (–0.02 to 0.11)		
Very high–level intervention × population mobility to visit parks and do leisure activities	0.02 (0.01 to 0.03)	0.05 (0.03 to 0.07)	0.09 (0.06 to 0.13)		–0.001 (–0.06 to 0.06)		
Low-level intervention × population mobility to workplaces (reference category)	N/A	N/A	N/A		N/A		
Intermediate-level intervention × population mobility to workplaces	0.01 (–0.01 to 0.03)	0.03 (–0.03 to 0.08)	0.03 (–0.10 to 0.16)		0.07 (–0.02 to 0.16)		
High-level intervention × population mobility to workplaces	0.02 (0.01 to 0.04)	0.01 (–0.01 to 0.03)	0.0004 (–0.02 to 0.02)		0.04 (–0.00^b^ to 0.08)		
Very high–level intervention × population mobility to workplaces	0.03 (0.01 to 0.04)	0.02 (–0.00^b^ to 0.03)	–0.0002 (–0.02 to 0.02)		0.04 (–0.00^b^ to 0.08)		

^a^Models were also adjusted by country income level; preparedness in epidemics (Index for Risk Management); COVID-19 type of transmission (ie, community transmission or local transmission); and COVID-19 testing and tracing policies (in days).

^b^These are values less than 0.005 in absolute numbers.

^c^N/A: not applicable.

#### Population Mobility Patterns and COVID-19 Spread for Latin America, the Caribbean, and Africa

When the analysis was stratified by Latin American, Caribbean, and African countries, specific trends in COVID-19 spread were shown due to distinct population’s mobility patterns (Table 3). As noted, the coefficients of the interaction between the populations’ mobility to parks and similar places and social distancing measures were consistent at the regional level, apart from the region of Africa. Specifically, when high-level and very high–level control policies were applied in comparison with those at a low level, only people’s mobility to parks and similar places was related with increased COVID-19 spread (exception was the African countries where the results were not significant; ie, Latin America, the Caribbean, and Africa: high-level interventions concurrently with mobility to parks and similar places *b*=0.05, 95% CI 0.02-0.07; very high–level interventions concurrently with mobility to parks and similar places *b*=0.05, 95% CI 0.03-0.07). Moreover, the interaction between populations’ mobility to workplaces and social distancing measures showed heterogeneity among the tested regions. However, apart from the global analysis, results were not significant for the Latin American, Caribbean, or African regions.

Finally, we conducted a sensitivity analysis to assess our inferences for large countries in terms of area extension (due to possible subnational mitigation policies) and to avoid a possible bias effect toward big country areas, taking into account our analytical sample of countries (n=179). For the sensitivity analysis, we fitted all models again for data on COVID-19 spread removing the 5 top countries with the largest area worldwide (ie, Russia, Canada, China, the United States, and Brazil) and the top 3 countries with the largest area regionally for Latin America and for Africa (Brazil, Angola, Argentina, etc). Again, the observed results remained in the same direction at the global and regional level, as was previously mentioned (data not shown in the text).

## Discussion

### Principal Findings

This study analyzed the geographical and temporal COVID-19 spread among LMICs in Latin America, the Caribbean, and Africa using the prospective space–time scan statistical methodology and the impact of real-time population mobility patterns during the implemented government interventions in the area of interest between January 21 and May 18, 2020. First, analyzing the current data with scan statistics at five prospective time periods, it was shown that virus spread was rapid and at alarming rates since March 15, where we detected 7 emerging COVID-19 clusters, which at May 15 had spread to 28, among the regions of Latin America, the Caribbean, and Africa. As governments decide their strategies in response to the pandemic, surveillance is of importance especially among LMICs that have limited resources at their disposal; hence, the prospective scan statistic could be used as a useful surveillance tool at the international, national, and subnational levels. Second, as presented in 4 country-specific examples, the prospective scan statistic showed high predictive validity with classic surveillance technics. Third, when the real-time mobility to parks, beaches, and other similar places as well as the mobility to workplaces were tested as individual factors, it was shown that these patterns were related with reduced COVID-19 spread. However, worldwide, the population movement to parks, beaches, and other similar places (although more reduced than the reference period) seemed to be related with increased virus spread with all levels (intermediate, high, very high) of government control policies activated (when compared with the low-level government control policies). Fourth, similar trends were shown for population mobility to workplaces when high and very high–level control policies (after comparing them with low-level government interventions) were implemented worldwide. Fifth, stratified analysis for the Latin American, Caribbean, and African regions showed a variety of patterns mostly following the entire samples’ tendency (ie, real-time mobility to parks when the social distancing measures were implemented). Governments are applying social and mobility restriction measures to slowdown the COVID-19 spread, but there is limited information about the real-time population mobility patterns, and based on our analysis, this information could help public health authorities to design effective strategies to slow down virus transmission.

The major strength of the prospective space–time scan methodology is the ability to add dynamically updated data sets and reapply the analysis to extract new emerging COVID-19 clusters, while it also has the ability to monitor the growing or shrinking COVID-19 evolution among initial detected clusters. Our analysis showed that Antigua and Barbuda had an emerging COVID-19 cluster with one of the highest RRs in Latin America and the Caribbean by the end of March 2020 and continues with a shrinking magnitude by mid-May. Similar trends were reported for Mexico and Chile. The same tool could be used subnationally among these countries to detect emerging clusters at the cross-national level (as has been done for the United States [10]). Regarding African regions, Djibouti, Mauritius, and Egypt showed growing and reducing magnitudes in COVID-19 spread from January 21 to May 15, 2020. This kind of information could be helpful to the relative stakeholders since it gives the opportunity to the public health authorities to evaluate constantly the effectiveness of the implemented mitigation and control strategies. Our comparative analysis between prospective scan and SIR modeling among 4 areas showed similar predictive results in virus spread. As has recently been reported, effective COVID-19 surveillance and monitoring need to include additional information on suspected, probable, and negative COVID-19 tests for a holistic understanding of COVID-19 transmission patterns [29], something that is often not possible for LMICs and could be marked as a barrier. Thus, future studies, data sets, and research funding are needed [30]. Health policy research showed that countries should not phase out social distancing policies until they establish strong systems that could effectively monitor the COVID-19 spread [15]. For this reason, at the early stages of the virus spread, the prospective scan methodology could serve as a useful public health surveillance tool especially among LMICs that are facing substantial limitations to monitor and detect virus transmission.

A lot of discussion has happened about the role of government interventions and control policies on COVID-19 spread. Until today, there is limited information on the impact of social restrictions and control policies at the global, regional, and national levels [15,25]. In Europe, the strictest government policy measures are related with less virus spread [31]. Targeted national health policies with effective screening and isolation tools as well as support systems are needed [15]. Based on that, real-time population mobility patterns during this intervention and social distancing period could be useful to stakeholders and policy makers to plan current and future public health–targeted strategies. Recent studies showed the important role of COVID-19–targeted strategies at the national level [32,33]. Moreover, this kind of information could serve among LMICs with limited resources in social distancing implementation, allowing them to plan targeted mobility control activities [34].

To date, although this kind of information is publicly available from well-known crowdsource applications, analyses at a national, regional, or global level are lacking. Our study used the publicly available crowdsource mobility information and showed that at the global level, when all kinds of government control policies were implemented, mobility to parks, beaches, and other leisure places was related with accelerated COVID-19 spread when compared with places where low-level control policies were effective. At a global level, peoples’ mobility for work was also related to increased virus spread when high-level and very high–level government interventions were active, after comparing with the reference category of low-level government interventions. However, at the regional level (LMICs among Latin America, the Caribbean, and Africa), the results were not significant, showing that the aforementioned relation is mainly driven from the rest of the areas around the world. At this point, it has to be noted that, when we did not take into account the interaction with governmental social distancing interventions, the aforementioned mobility patterns were related with reduced virus transmission.

During the period that a region is facing increased dynamics of COVID-19 spread, social activities and engagement are associated with increased risk for virus spread [35]. For this reason, the WHO and other public health organizations are recommending avoiding crowded conditions [36]. Recent data from the United States showed that lower mobility to workplace and retail locations is related with lower virus transmission [35]. Our entire sample analysis showed a positive relation between mobility to places like parks and workplaces while governments applied social distancing measures. Similar findings were also reported by other researchers for the United States [35]. In addition, our regional stratified analysis showed consistent findings with the global one, except from the region of Africa. Taking into account that human mobility is a complicated concept (and at this point is analyzed collectively by using crowdsource data), we may hypothesize that individuals’ behavior (use of face mask) [32], people’s dynamic network [33] when visiting these places, and seasonality could be potential explaining mechanisms of virus spread [35,37]. The aforementioned findings could guide stakeholders on specific social distancing implementations and enforcement planning [38]. In the past months, countries are introducing various nonpharmaceutical intervention strategies in their local health policy agendas. These results can be used as *roadmap* indicators for specific social distancing planning. Targeted implemented policies could lead to further suppressed levels of virus spread, with less negative effects on the economy and citizens [39]. To this extent, a recent study noted that, during the phasing out of government social distancing policies, higher mobility at workplaces was correlated with increasing virus spread [40].

### Effectiveness of the Current Measures and Current and Future Challenges

To date, countries have adopted divergent restriction strategies to suppress and halt COVID-19 transmission. Stricter social distancing policies seem effective in suppressing virus spread [31]. Differences in innovative surveillance techniques, virus transmission monitoring, COVID-19 cases tracing, systematic population testing, and isolating practices have been shown [15]. Regions with previous experiences in infectious diseases (eg, severe acute respiratory syndrome) have invested in their public health care system’s reformation to efficiently handle the current outbreak [15]. Western societies (eg, the European Union and the United States) seem to lack this kind of planning [41]. In addition, recent studies showed that COVID-19 spread could be more rapid among more prosperous countries [15,42]. Countries need to organize their health systems [15,41], establishing effective infectious diseases and crisis management planning [43] (eg, enhance monitoring techniques and screening tools) to prevent virus spread in the community. Future longitudinal studies may be needed to better describe the relation of real-time mobility data with COVID-19 transmission.

### Strengths and Limitations

This is among the first studies using COVID-19 prospective surveillance analysis among LMICs, exploring COIVD-19 spread in relation with real-time population mobility patterns. However, this study shares common limitations with previous studies of this kind [17,25]. Specifically, there were challenges in capturing uncertainty (completeness of the WHO COVID-19 data set or government interventions being announced on one day but only being applied after several days) and lags in data availability, which may not fully capture temporal trends of COVID-19 spread. We extracted only mobility patterns from smartphones using Google software to ensure homogeneity of the used information. In addition, this study had only the ability to analyze data from regions in which mobile phone information was obtainable [35]. For example, the use of crowdsourcing digital data for the extraction of real-time population trends through mobile phones may be limited particularly in Africa (data for selected African countries reported that about one-third of adults own smartphones) [44]. This may have altered the findings of this study. In addition, certain large countries applied subnational control policies at different time points, which could have affected our findings. In that manner, we applied a sensitivity analysis excluding large countries from the global and regional sample, and testing whether those countries had an impact on the inferential analyses. The applied aforementioned analysis showed that the results remained similar. Additionally, our investigation focused on data variations in the COVID-19 spread from January to May 2020. Therefore, the results of this paper should be interpreted with caution, as they only relate to the underlying data collection conditions and period. As COVID-19 is an infection with dynamic transmission and all the variables we use may variate in the future, we do not think it would be appropriate to make conclusions beyond May, as further data and analysis would be required. To this extent, it should also be noted that this mixed model analysis assumes that the impact of each relative mobility pattern change has the equal relative impact among countries and across time (as an additional adjustment to this extent was not possible). Next, although our study adjusted for various confounders, we could not consider physical distancing recommendations (ie, 1 or 2 meters) or other precautionary measures and conditions due to lack of data. In addition, some of the mobility variables used in this analysis (eg, parks) may be also affected from weather seasonality. The prospective space–time scan statistic used case data for confirmed cases, so suspected and probable cases were not considered due to the unavailability of the WHO COVID-19 data set. In addition, the prospective scan methodology does not allow for adjustment of age and other covariates. These limitations may alter the true magnitude of the COVID-19 spread as presented by using the prospective scan statistic.

### Conclusions

We used publicly available WHO daily reports to identify emerging space–time clusters of COVID-19 at the country level among Latin America, the Caribbean, and Africa for five separate time periods. It was shown that the prospective scan is a tool that LMICs could use to detect emerging clusters and implement specific control policies and interventions to slowdown COVID-19 transmission. In addition, we found that different kinds of real-time population mobility patterns were related with different magnitudes of COVID-19 spread worldwide. The findings of this study give insights that may help in COVID-19 screening and detection strategies as well as in government–specific COVID-19 control planning.

## Appendix

Multimedia Appendix 1 Supplementary material.

## Abbreviations

INFORM: Index for Risk Management
LIC: low-income country
LMIC: low-income and middle-income country
NB: negative binomial
RR: relative risk
SIR: standardized incidence ratio
WHO: World Health Organization
