# Supplementary material for: Estimating the COVID-19 Spread Through Real-time Population Mobility Patterns: Surveillance in Low- and Middle-Income Countries
Source: J Med Internet Res. 2021 Jun 14;23(6):e22999. doi: 10.2196/22999 (PMC8204939; doi:10.2196/22999)
Supplement: Multimedia Appendix 1 [file jmir_v23i6e22999_app1.docx]

**Estimating the COVID-19 spread through real-time population mobility patterns: surveillance in Low- and Middle- income countries**

**Figure S1.** Spatial distribution of emerging space-time clusters of COVID-19 at country level from January 21^st^ -March 15^th^, 2020, for Latin America and Caribbean.………………………3

**Figure S2.** Spatial distribution of emerging space-time clusters of COVID-19 at country level from January 21^st^ -March 15^th^, 2020, for Africa…………………………………………………………………………………………..4

**Figure S3.** Spatial distribution of emerging space-time clusters of COVID-19 at country level from January 21^st^ -March 31^st^, 2020, for Latin America and Caribbean……............................5

**Figure S4.** Spatial distribution of emerging space-time clusters of COVID-19 at country level from January 21^st^ -March 31^st^, 2020, for Africa………………………………………………..6

**Figure S5.** Spatial distribution of emerging space-time clusters of COVID-19 at country level from January 21^st^ – April 15^th^, 2020, for Latin America and Caribbean……………………....7

**Figure S6.** Spatial distribution of emerging space-time clusters of COVID-19 at country level from January 21^st^ – April 15^th^, 2020, for Africa………………………………………………..8

**Figure S7.** Spatial distribution of emerging space-time clusters of COVID-19 at country level from January 21^st^ – April 30^th^, 2020, for Latin America and Caribbean……………………...9

**Figure S8.** Spatial distribution of emerging space-time clusters of COVID-19 at country level from January 21^st^ – April 30^th^, 2020, for Africa……………………………………………...10

**Figure S9.** Spatial distribution of emerging space-time clusters of COVID-19 at country level from January 21^st^ – May 15^th^, 2020, for Latin America and Caribbean………………….…..11

**Figure S10.** Spatial distribution of emerging space-time clusters of COVID-19 at country level from January 21^st^ – May15^th^, 2020, for Africa……………………………………………….12

**Figure S11**. Comparison and correlation of relative risk (RR) and standardized incidence ratios (SIR) ratios for Brazil, Peru, Uganda and Nigeria from 21^st^ – May 15^th^, 2020…………………………………………………………………………………………..13

**Figure S12.** COVID-19 daily new cases and real-time population mobility changes in Africa and Latin America and the Caribbean……………………………………………………..14-15

Figure S1. Spatial distribution of emerging space-time clusters of COVID-19 at country level from January 21^st^ -March 15^th^, 2020, for Latin America and Caribbean.


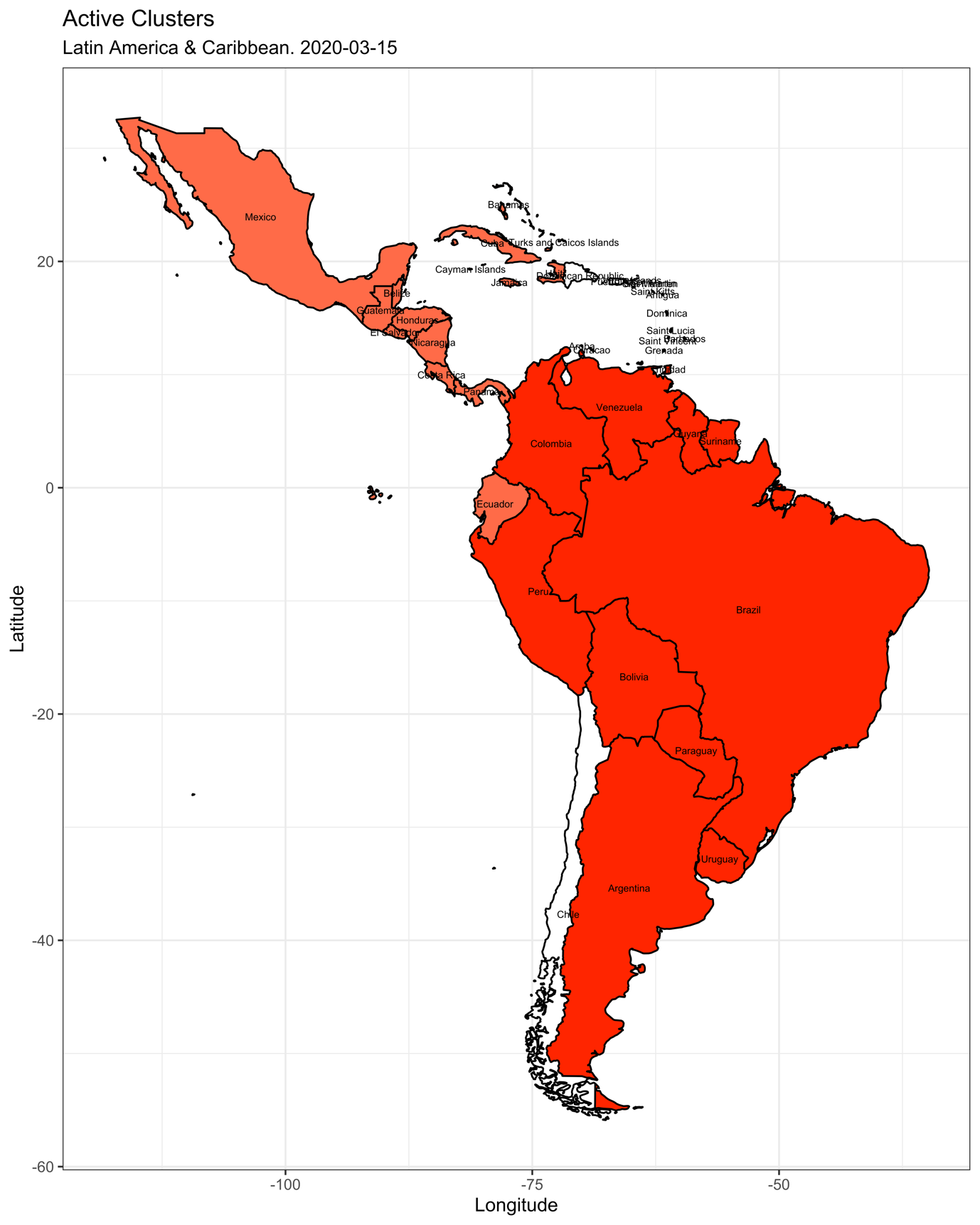


The red coloring scale shows the importance of active country-level clusters. Colors span from white, grey, and then red. The redder the more important.  White color indicates a non-statistically significant cluster (Gumbel p-value>0.05).

Figure S2. Spatial distribution of emerging space-time clusters of COVID-19 at country level from January 21^st^ -March 15^th^, 2020, for Africa.


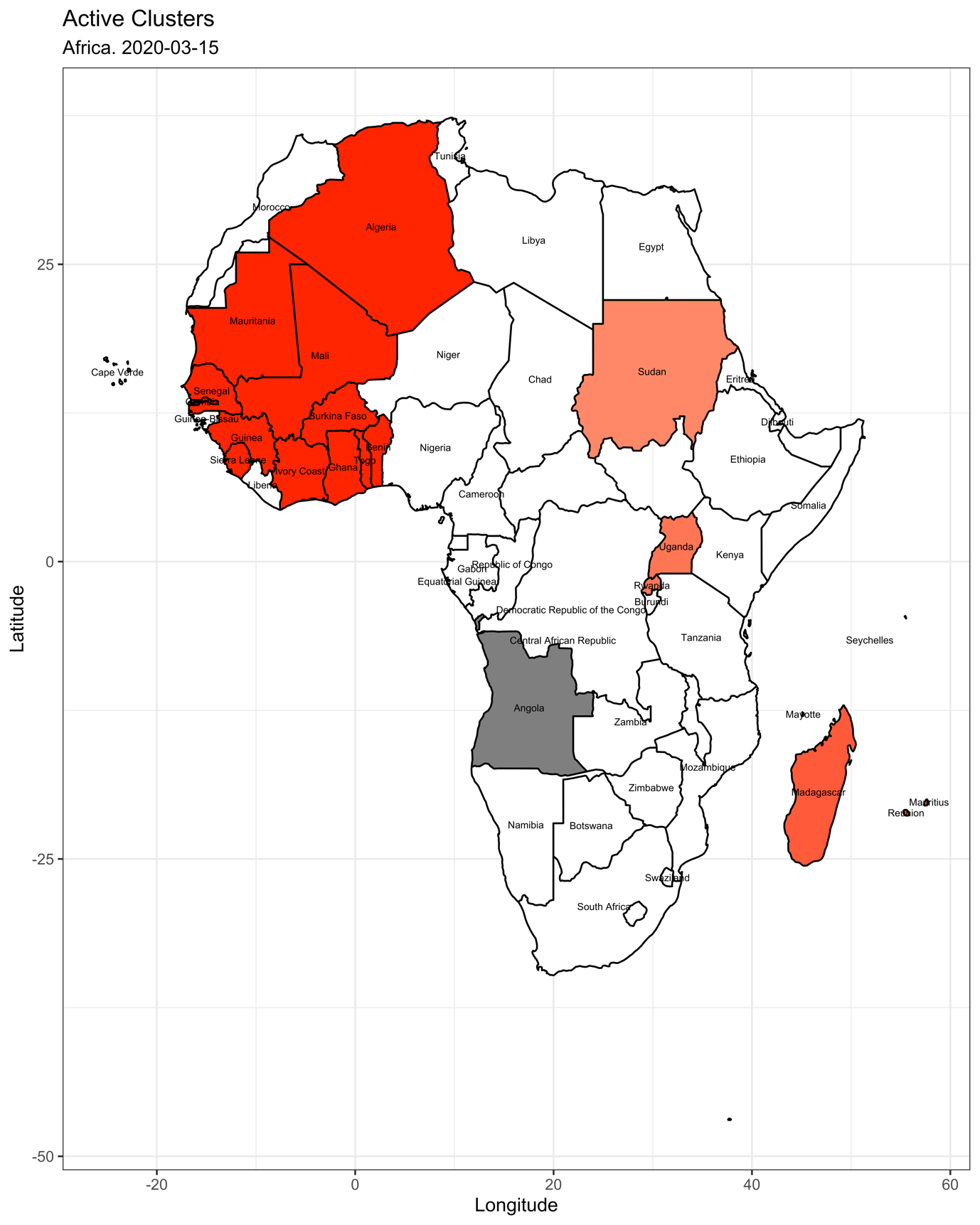


The red coloring scale shows the importance of active country-level clusters. Colors span from white, grey, and then red. The redder the more important.  White color indicates a non-statistically significant cluster (Gumbel p-value>0.05).

Figure S3. Spatial distribution of emerging space-time clusters of COVID-19 at country level from January 21^st^ -March 31^st^, 2020, for Latin America and Caribbean.


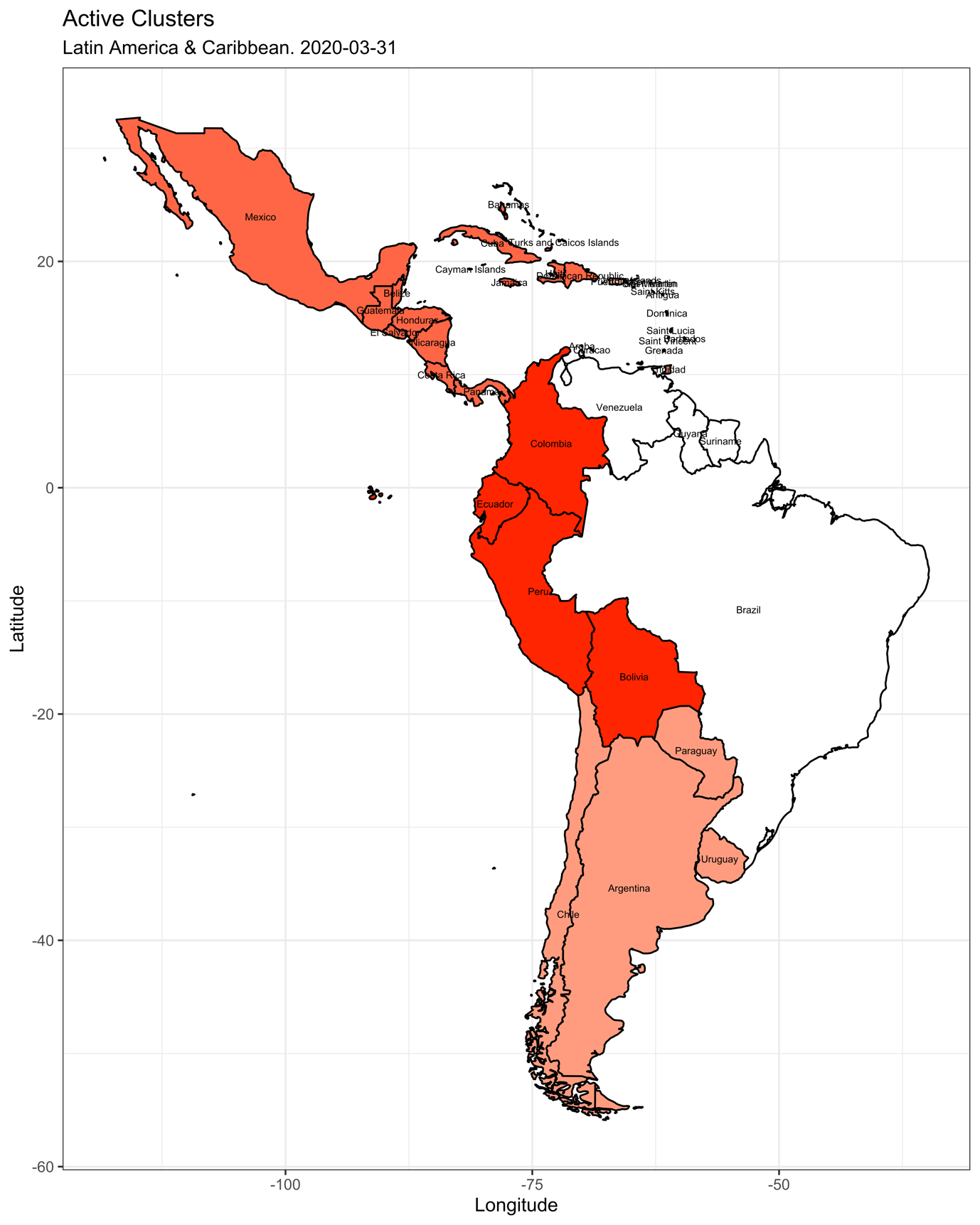


The red coloring scale shows the importance of active country-level clusters. Colors span from white, grey, and then red. The redder the more important.  White color indicates a non-statistically significant cluster (Gumbel p-value>0.05).

Figure S4. Spatial distribution of emerging space-time clusters of COVID-19 at country level from January 21^st^ -March 31^st^, 2020, for Africa.


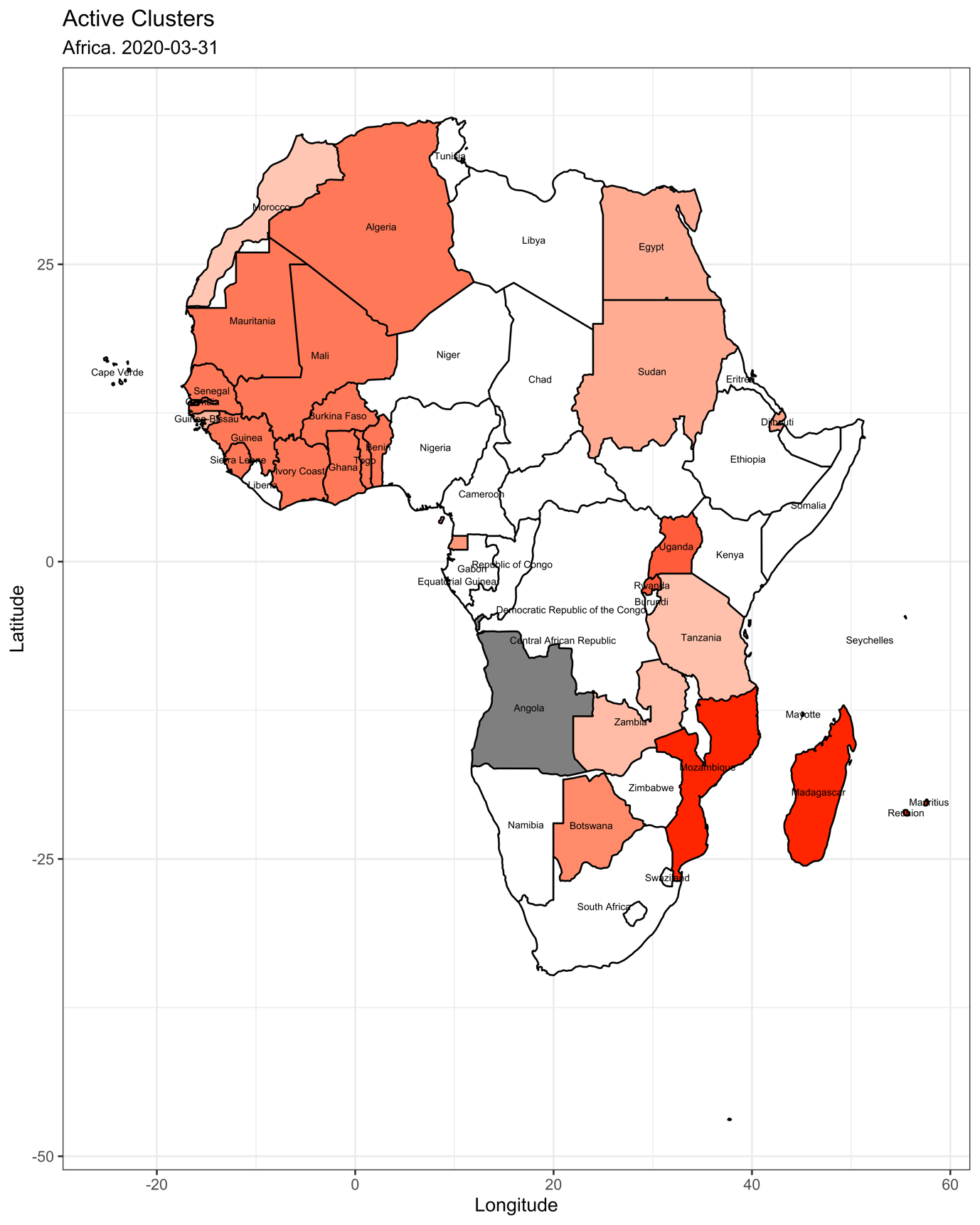


The red coloring scale shows the importance of active country-level clusters. Colors span from white, grey, and then red. The redder the more important.  White color indicates a non-statistically significant cluster (Gumbel p-value>0.05).

Figure S5. Spatial distribution of emerging space-time clusters of COVID-19 at country level from January 21^st^ -April 15^th^, 2020, for Latin America and Caribbean.


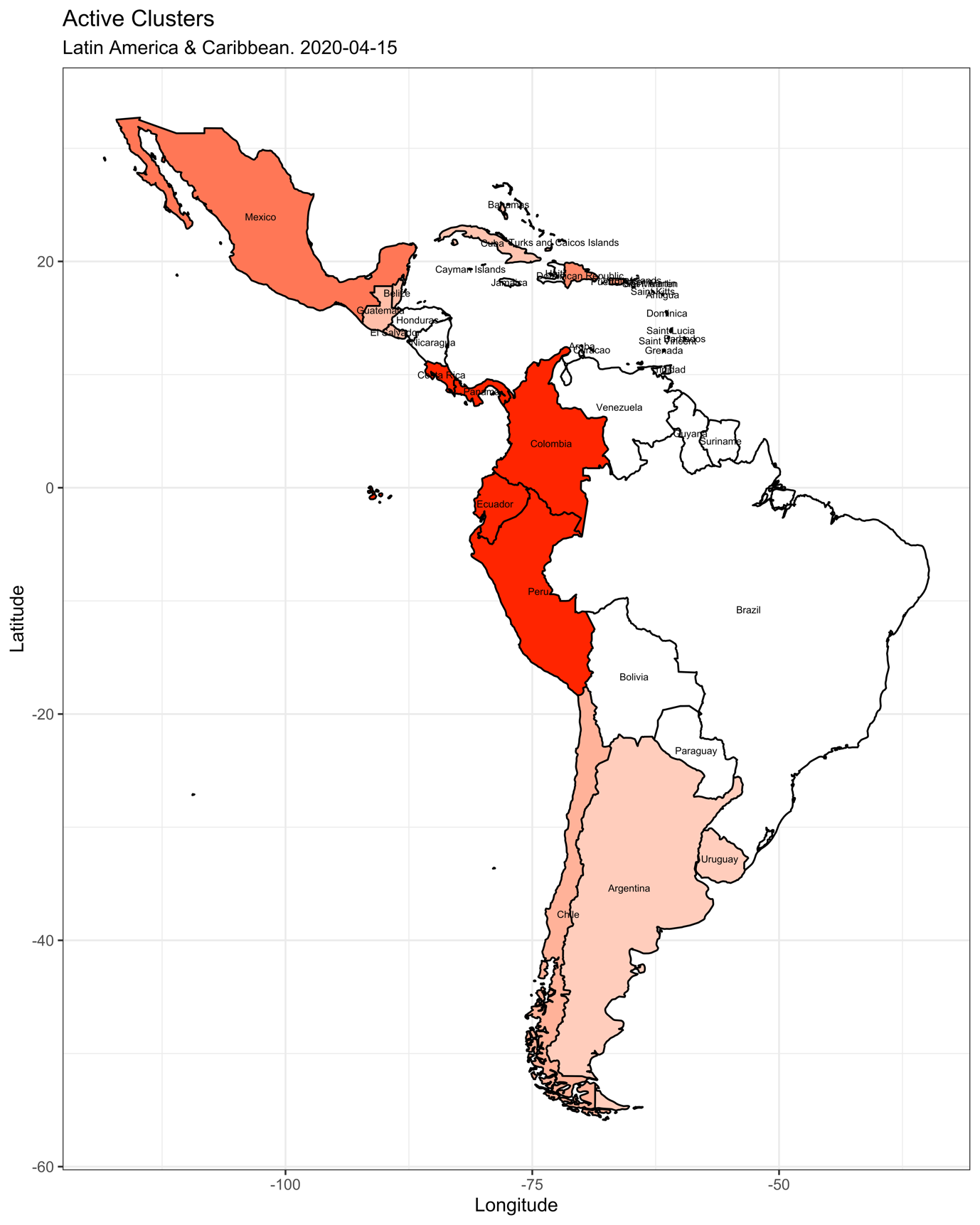


The red coloring scale shows the importance of active country-level clusters. Colors span from white, grey, and then red. The redder the more important.  White color indicates a non-statistically significant cluster (Gumbel p-value>0.05).

Figure S6. Spatial distribution of emerging space-time clusters of COVID-19 at country level from January 21^st^ -April 15^th^, 2020, for Africa


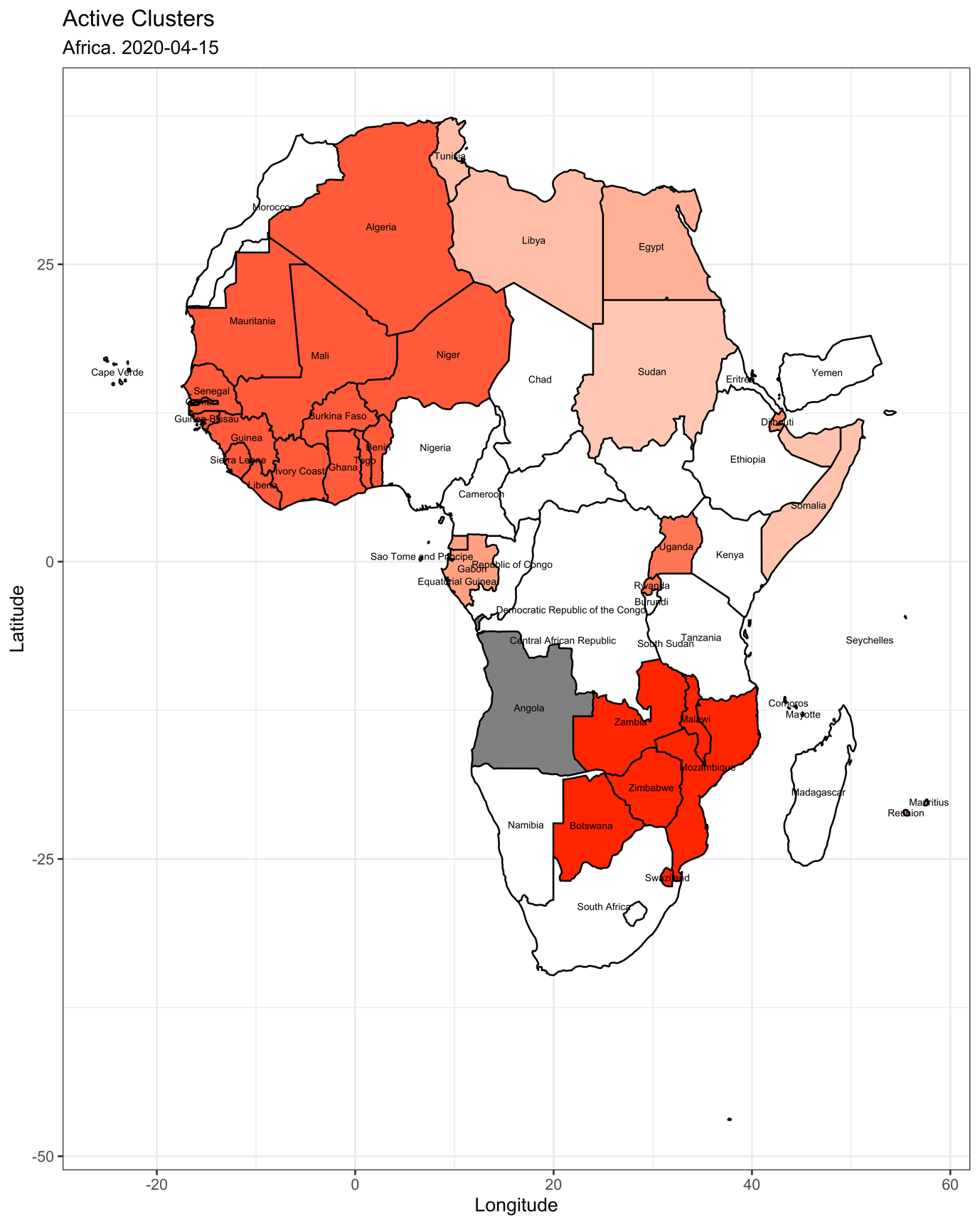


The red coloring scale shows the importance of active country-level clusters. Colors span from white, grey, and then red. The redder the more important.  White color indicates a non-statistically significant cluster (Gumbel p-value>0.05).

Figure S7. Spatial distribution of emerging space-time clusters of COVID-19 at country level from January 21^st^ -April 30^th^, 2020, for Latin America and Caribbean


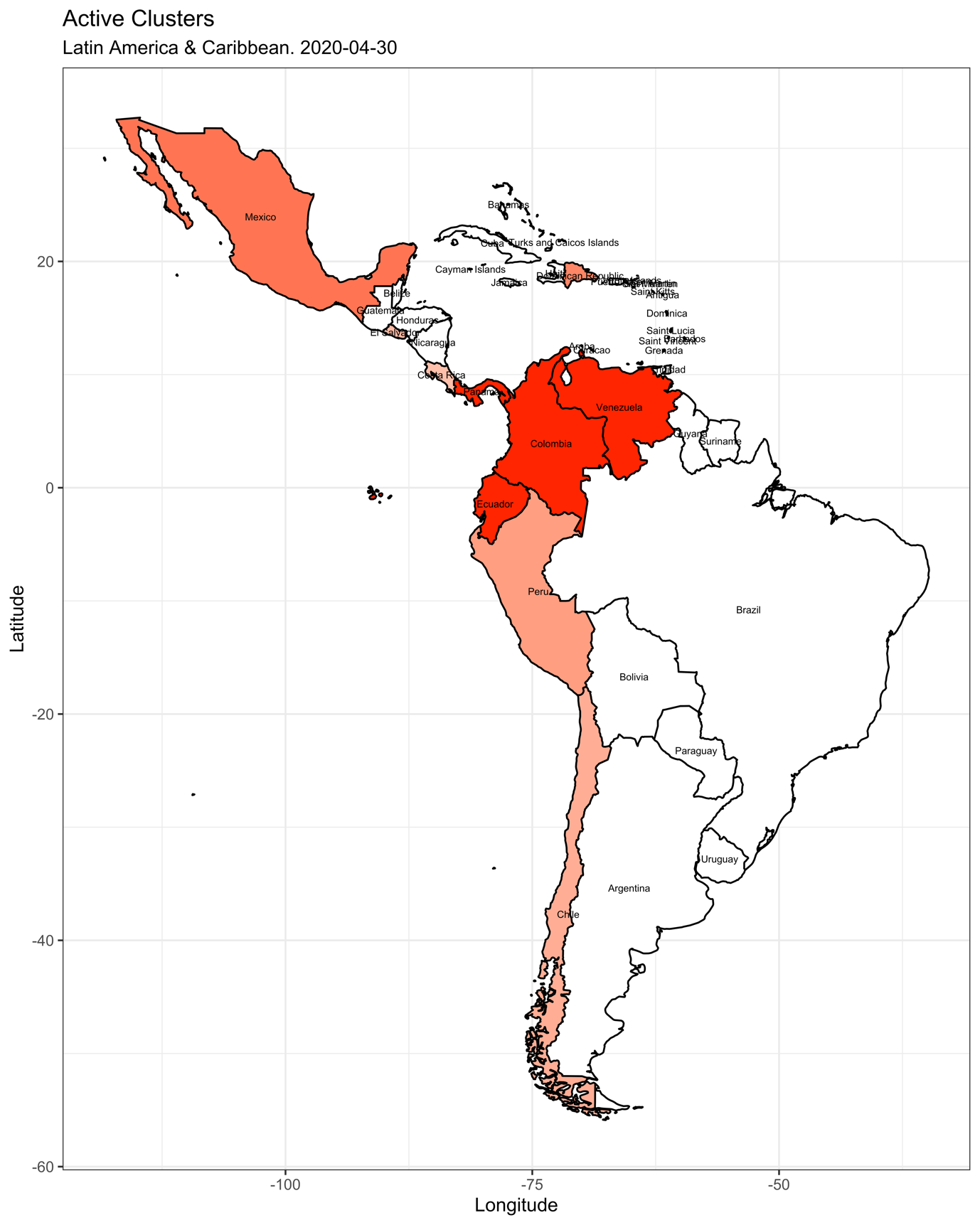


The red coloring scale shows the importance of active country-level clusters. Colors span from white, grey, and then red. The redder the more important.  White color indicates a non-statistically significant cluster (Gumbel p-value>0.05).

Figure S8. Spatial distribution of emerging space-time clusters of COVID-19 at country level from January 21^st^ -April 30^th^, 2020, for Africa


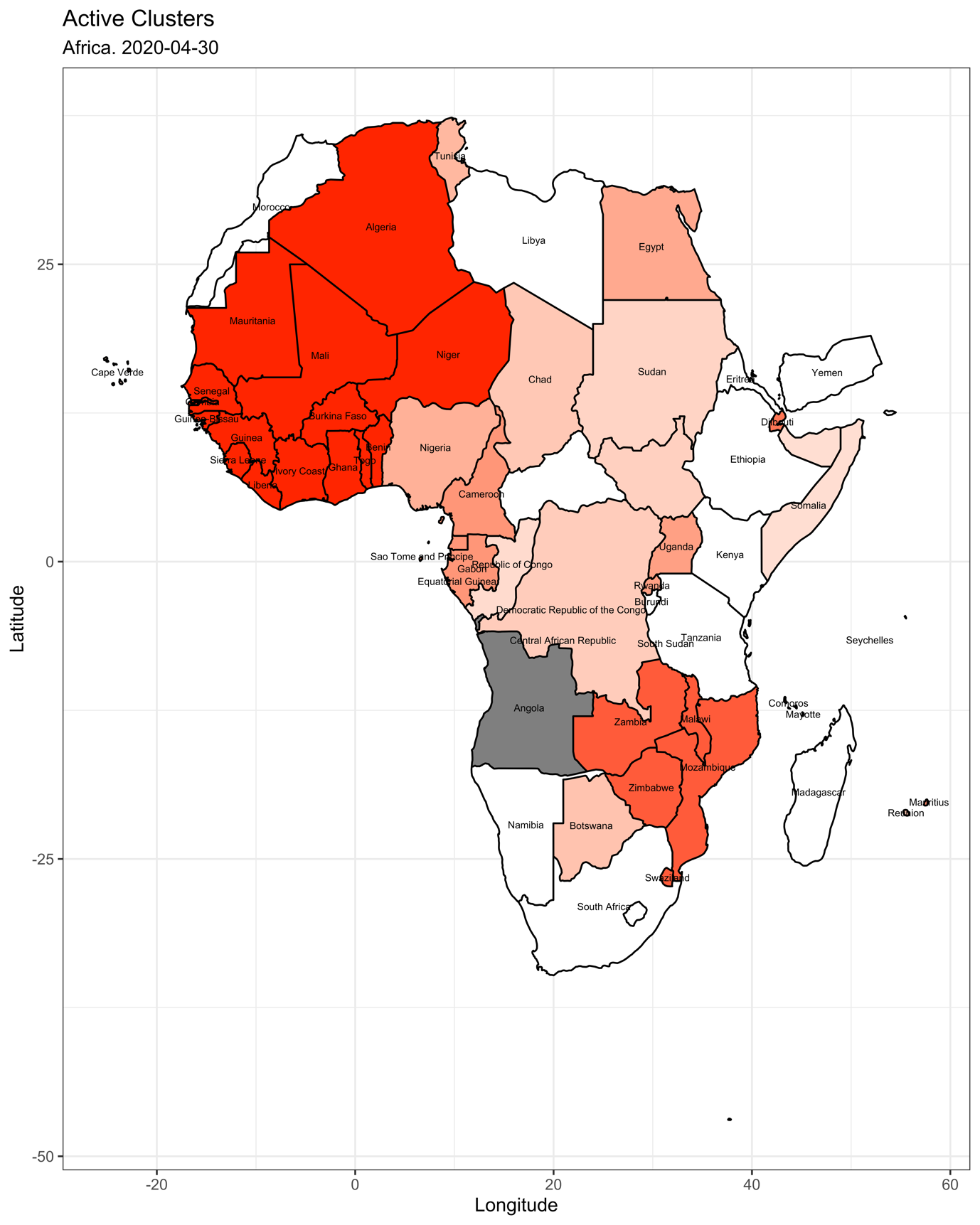


The red coloring scale shows the importance of active country-level clusters. Colors span from white, grey, and then red. The redder the more important.  White color indicates a non-statistically significant cluster (Gumbel p-value>0.05).

Figure S9. Spatial distribution of emerging space-time clusters of COVID-19 at country level from January 21^st^ -May 15^th^, 2020, for Latin America and Caribbean


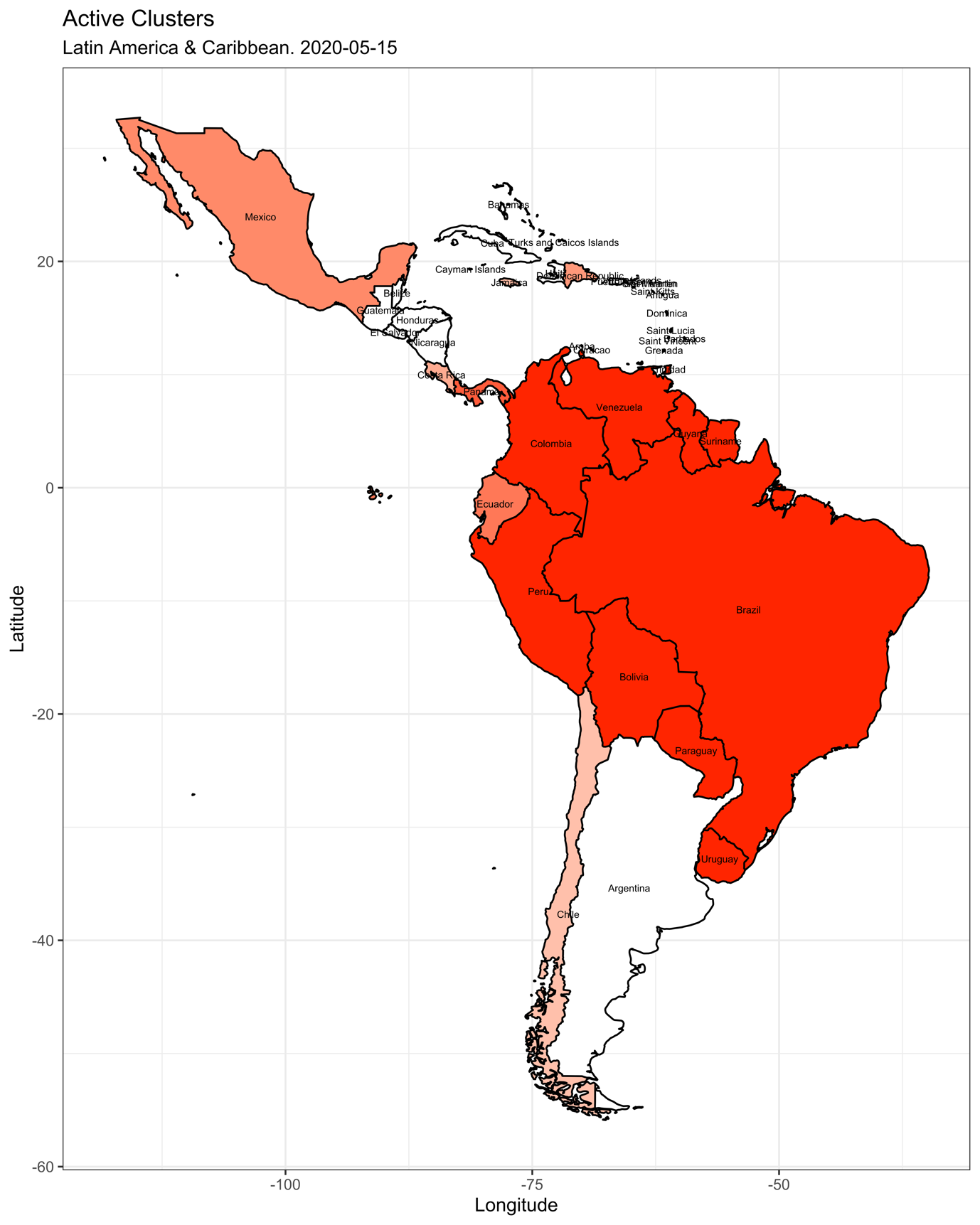


The red coloring scale shows the importance of active country-level clusters. Colors span from white, grey, and then red. The redder the more important.  White color indicates a non-statistically significant cluster (Gumbel p-value>0.05).

Figure S10. Spatial distribution of emerging space-time clusters of COVID-19 at country level from January 21^st^ -May 15^th^, 2020, for Africa


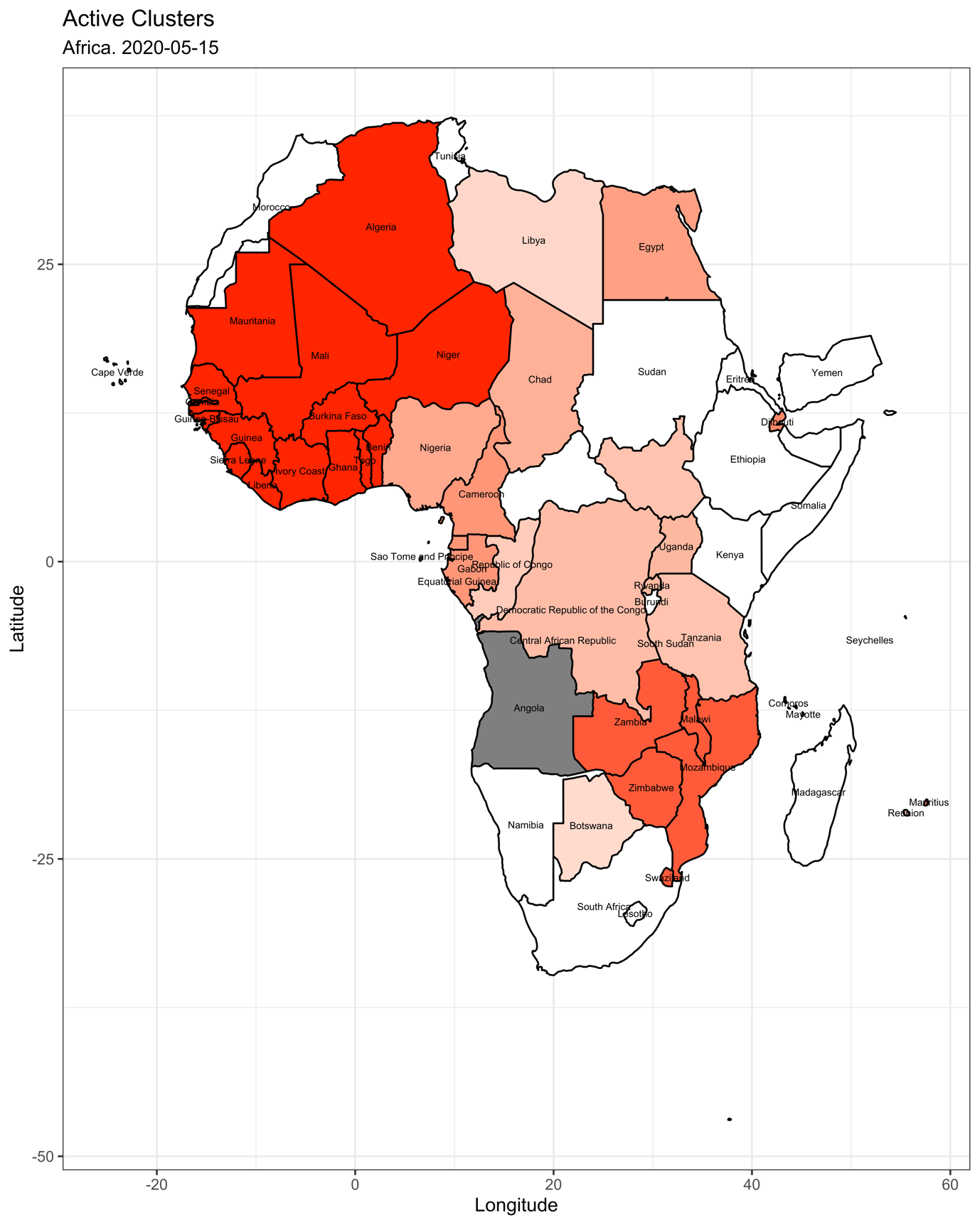


The red coloring scale shows the importance of active country-level clusters. Colors span from white, grey, and then red. The redder the more important.  White color indicates a non-statistically significant cluster (Gumbel p-value>0.05).

Figure S11. Comparison and correlation of prospective scan relative risk (RR) and SIR ratios for Brazil, Peru, Uganda, and Nigeria from 21^st^ – May 15^th^, 2020.


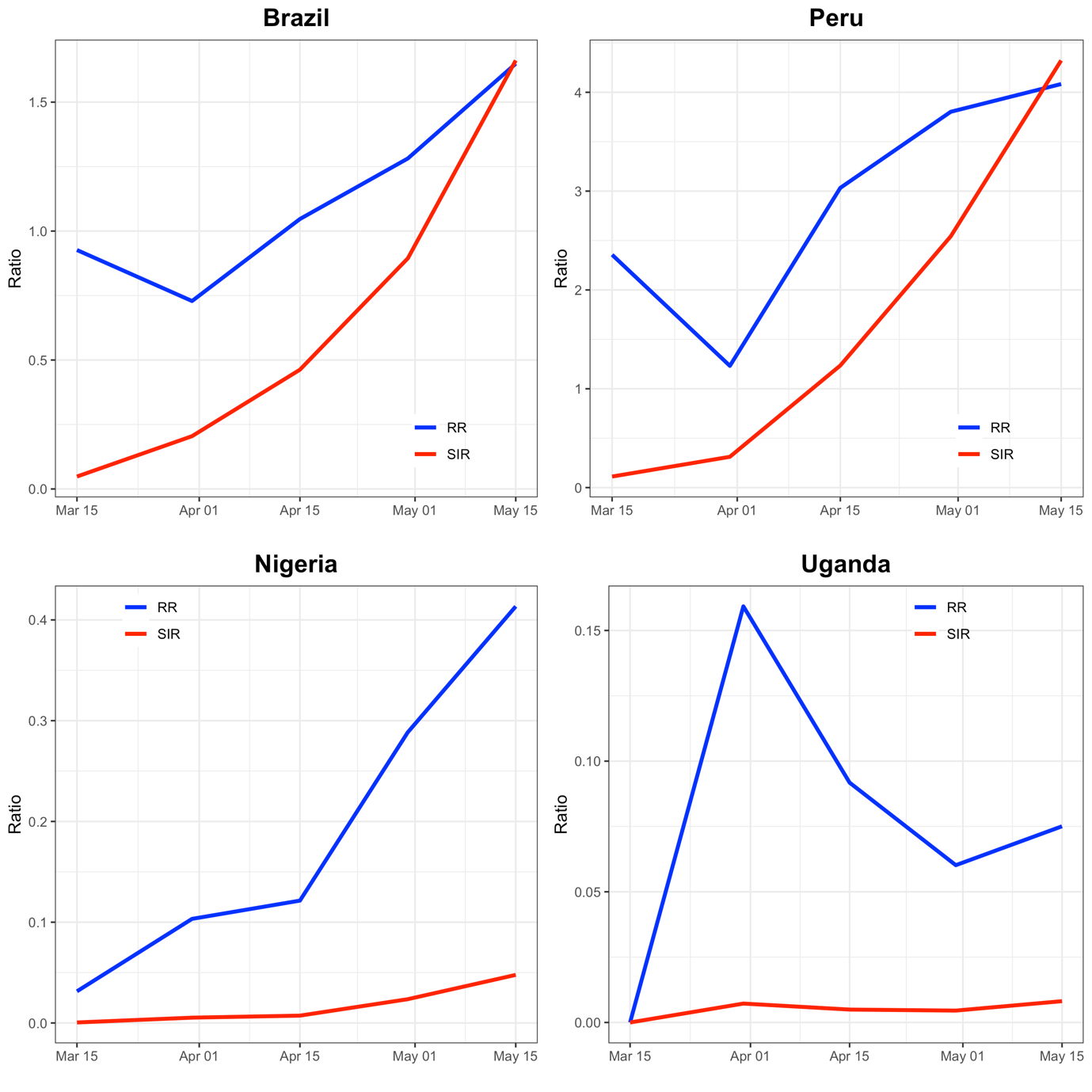
The blue line pictures the evolution of relative risk (RR) ratios from the prospective scan approach, while the red line pictures the evolution of COVID-19 ratios based on SIR estimation. Pearson correlations were strong for both four countries (Uganda; Rho= 0.78, P=0.04, Nigeria; Rho=0.98, P=0.002, Brazil; Rho=0.95, P=0.01 and Peru; Rho=0.86, P=0.03).

SIR: standardized incidence ratios

Figure S12. COVID-19 daily new cases per 100,000 population and real-time population mobility changes in worldwide, in Africa and in Latin America and the Caribbean


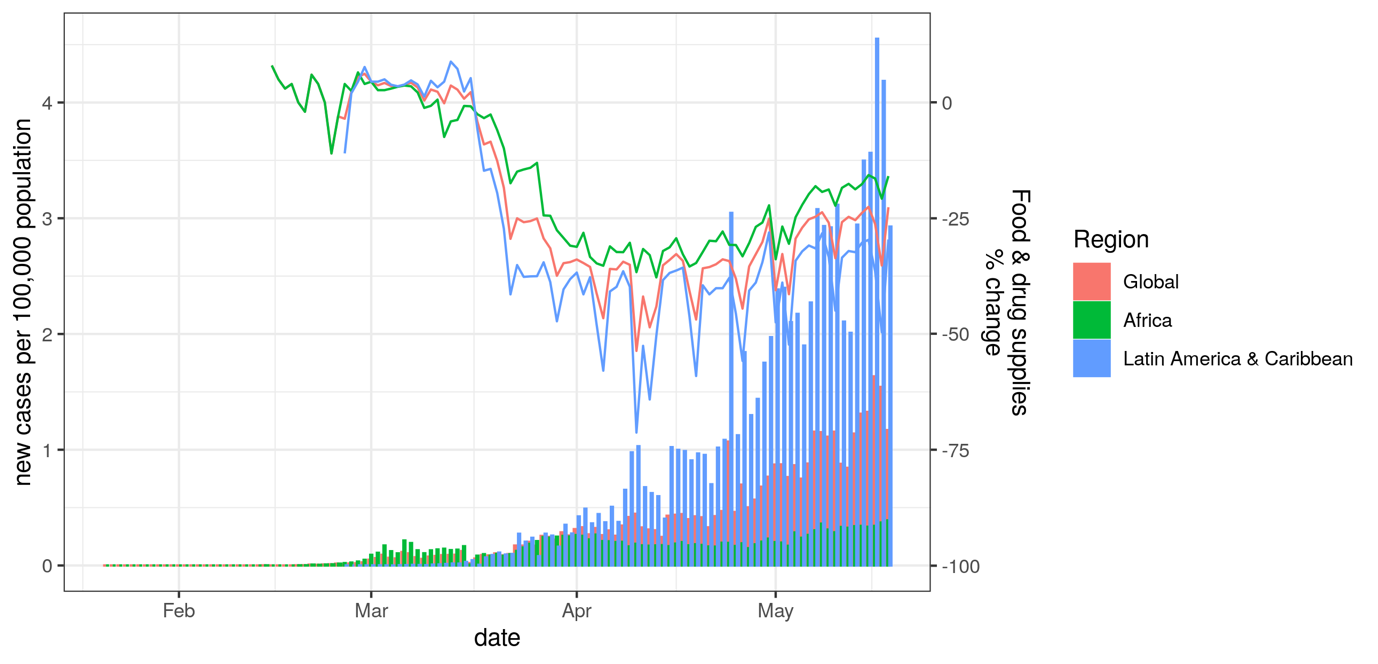


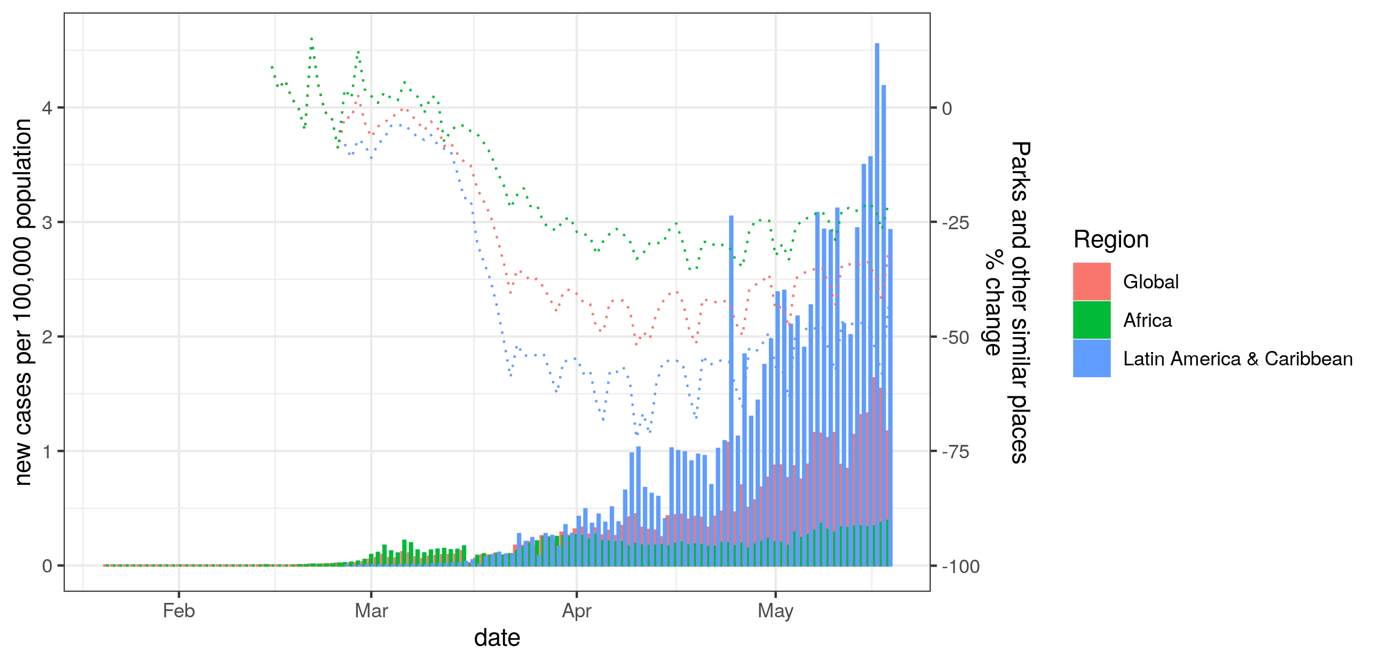


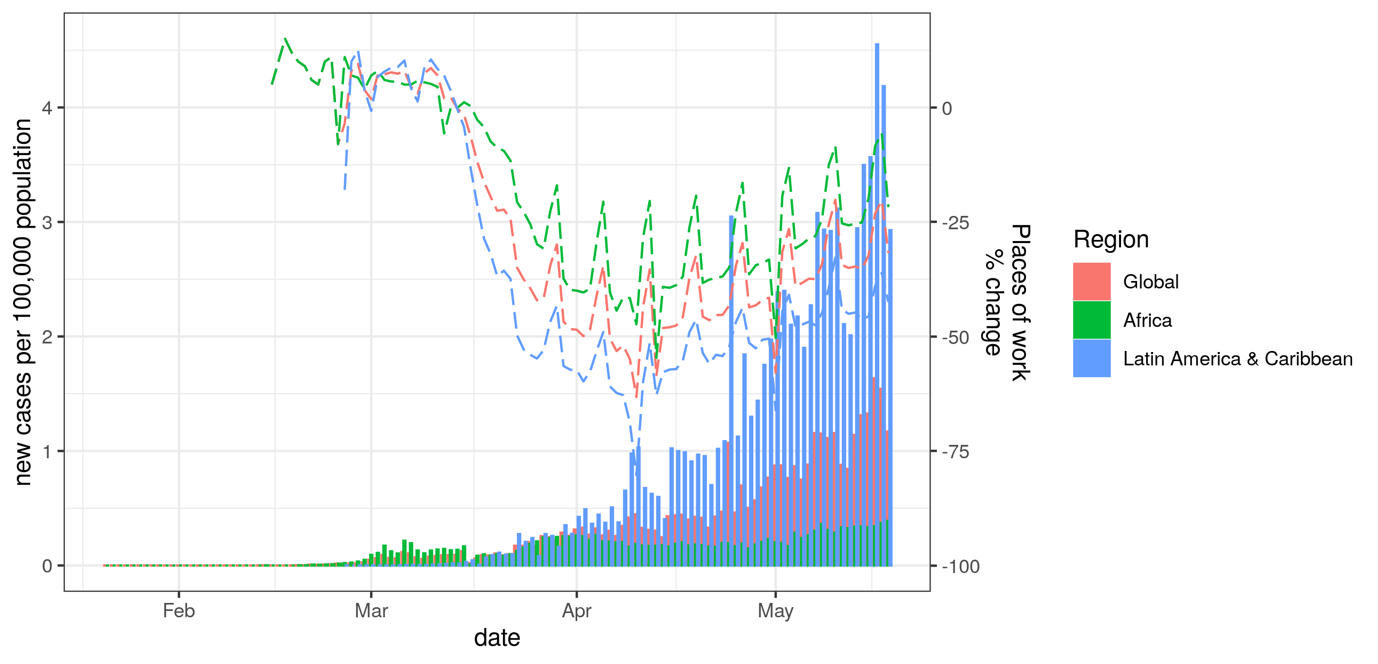


The line diagram shows the % of the change in population visits and length of stay at different places compared to a baseline period (as baseline period is defined as the period between January 3rd and February 6th, 2020), while the bar graph presents the daily new COVID-19 cases per 100,000
